# ﻿Description of three new species previously identified as *Stolephorusbengalensis* (Dutt & Babu Rao, 1959) or *Stolephorusinsularis* Hardenberg, 1933 and a re-description of *S.bengalensis* (Chordata, Osteichthyes, Clupeiformes, Engraulidae)

**DOI:** 10.3897/zookeys.1121.84171

**Published:** 2022-09-15

**Authors:** Harutaka Hata, Sébastien Lavoué, Hiroyuki Motomura

**Affiliations:** 1 National Museum of Natural History, Smithsonian Institution, 10; 2 th; 3 and Constitution Ave, NW Washington, DC 20560, USA; 4 School of Biological Sciences, Universiti Sains Malaysia, 11800, Penang, Malaysia; 5 The Kagoshima University Museum, 1-21-30 Korimoto, Kagoshima 890-0065, Japan

**Keywords:** Actinopterygii, Clupeomorpha, phylogenetics, *Stolephorustri*, taxonomy

## Abstract

Examination of numerous specimens characterised by predorsal scute, long maxilla, indented preopercle and pelvic scute lacking a spine and previously identified as *Stolephorusbengalensis* (Dutt & Babu Rao, 1959) or *Stolephorusinsularis* Hardenberg, 1933, revealed four distinct species, true *S.bengalensis* (distributed from the Bay of Bengal to Pakistan) and three new species, viz., *Stolephoruseldorado***sp. nov.** (Taiwan to Java, Indonesia), *Stolephorusdiabolus***sp. nov.** (Strait of Malacca, from Penang , Malaysia, to Singapore) and *Stolephoruseclipsis***sp. nov.** (Bintan Island, Riau Archipelago, Indonesia). Characters separating the four species include numbers of gill rakers on each gill arch and vertebrae and pelvic fin and dorsal-fin ray lengths. Two molecular markers (mitochondrial cytochrome *b* and cytochrome oxidase I genes) demonstrated the distinction of three of the species examined morphologically and enabled a reconstruction of their phylogenetic relationships. Each species was genetically divergent from the others by 3.5%–7.7% mean uncorrected distance in the mitochondrial cytochrome oxidase I gene.

## ﻿Introduction

**Figure 1.**
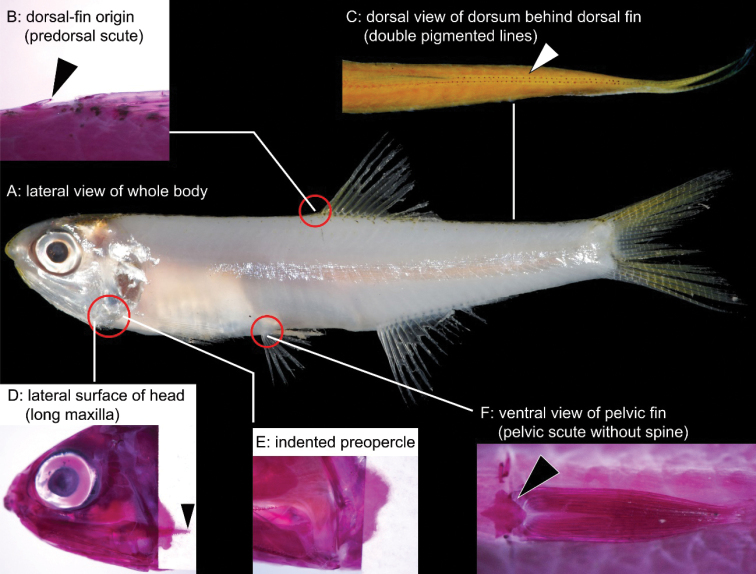
Diagnostic characters of species previously identified as *Stolephorusbengalensis***A** lateral view of whole body **B** dorsal-fin origin (triangle indicates predorsal scute, located just anterior to dorsal-fin origin) **C** dorsal view of dorsum behind dorsal fin (triangle indicates paired dark lines) **D** lateral surface of head (triangle indicates posterior tip of maxilla, posteriorly well beyond posterior margin of pre-opercle) **E** pre-opercle with concave posterior margin (supramaxilla removed) and **F** ventral view of pelvic fin (triangle indicates pelvic scute, lacking spine) (**A** KAUM–I. 94521, paratype of *S.eldorado* sp. nov. in fresh condition, 43.4 mm SL, Ha Long Bay, northern Vietnam **B, E, F** KAUM–I. 113148, paratype of *S.eldorado* sp. nov., 55.3 mm SL, Ke-tzu-liao, south-western Taiwan **C** ZUMT 62056, paratype of *S.diabolus* sp. nov., 38.4 mm SL, Singapore **D** KAUM–I. 94509, paratype of *S.eldorado* sp. nov., 41.4 mm SL, Ha Long Bay, northern Vietnam) (**B, D, E** and **F** alizarin stain).

The anchovy genus *Stolephorus* Lacepède, 1803 (Teleostei: Clupeiformes: Engraulidae), diagnosed by the presence of prepelvic scutes and an embedded urohyal and lack of postpelvic scutes, currently includes 37 valid species that preferentially inhabit marine and/or estuarine waters in the Indo-Pacific region (Wongratana 1983, 1987a, b; Whitehead et al. 1988; Wongratana et al. 1999; Kimura et al. 2009; Hata and Motomura 2018a, b, c, d, e, 2021a, b, c, 2022; Hata et al. 2019, 2020a, b, 2021; Gangan et al. 2020). Amongst them, species with a predorsal scute, paired dark lines on the dorsum behind the dorsal fin, a long maxilla (posterior tip well beyond the preopercle posterior margin), the preopercle posterior margin concave and pelvic scute without a posteriorly projecting spine (Fig. 1) are regarded as *Stolephorusinsularis* Hardenberg, 1933 by Whitehead et al. (1988), who reviewed the genus. Hata et al. (2019) revised the taxonomy of seven nominal species of *Stolephorus*, treating Whitehead et al.’s (1988)*S.insularis* as *Stolephorusbengalensis* (Dutt & Babu Rao, 1959) and regarding the nominal species *S.insularis* as a junior synonym of *Stolephorustri* (Bleeker, 1852). However, subsequent re-examination of specimens, identified as *S.bengalensis*, in fact revealed the presence of four species.

The aim of this study is to re-describe *S.bengalensis* and describe three new species of *Stolephorus* from specimens previously regarded as *S.insularis* or *S.bengalensis*. In addition to the morphological comparisons, complete mitochondrial cytochrome *b* gene and partial mitochondrial cytochrome oxidase I (COI) gene sequences from 31 specimens were used to estimate the genetic distinction of three of the latter (the fourth species unavailable) plus one unidentified, but related species from Segara Anakan Lagoon, Central Java, Indonesia (Nuryanto et al. 2017).

## ﻿Materials and methods

Counts and proportional measurements followed Hata and Motomura (2017). Counts of fin rays and vertebrae followed Hubbs and Lagler (1947), the last two rays of dorsal and anal fins being counted separately, unless they originated from the same base, in which case they were counted as one ray. Vertebrae counts includes urostyle. All measurements were made with digital calipers to the nearest 0.01 mm. “Pelvic scute” refers to a scute joined to the pelvic girdle and “prepelvic scute”, “postpelvic scute” and “predorsal scute” to hard spine-like scutes anterior to the pelvic fin, posterior to the pelvic fin and just anterior to the dorsal-fin origin, respectively. Osteological characters, including vertebral counts, were determined from radiographs of 32, 2, 14 and 45 specimens of *S.bengalensis*, *S.diabolus* sp. nov., *S.eclipsis* sp. nov. and *S.eldorado* sp. nov., respectively. Abbreviations are as follows – SL: standard length; HL: head length; and UGR, LGR and TGR: rakers on upper limb, lower limb and total gill rakers, respectively, with associated numbers indicating the specific gill arch. Institutional codes generally follow Sabaj (2020). USMFC stands for Universiti Sains Malaysia Fish Collection, School of Biological Sciences, Penang, Malaysia.

The mitochondrial genotypes of 31 specimens comprising three (out of four) species of *Stolephorus* examined in this study, plus one closely related, but unidentified species, were compared using the complete (1140 base pairs [bp]) cytochrome *b* gene and partial (648 bp) COI gene. The cytochrome *b* gene sequences were published in Hata et al (2019; 2020b) and are available in GenBank (Table 1). The COI gene was newly sequenced for 19 specimens of *S.eldorado*, including the holotype and several paratypes (Table 1) and the resulting data combined with COI sequences (available in GenBank) of *S.diablocus* (two specimens from West Peninsular Malaysia, including the holotype), *S.bengalensis* (eight specimens from India), *S.eldorado* (one specimen from China; Pang et al. 2019) and a single specimen of an unidentified *Stolephorus* species (from Segara Anakan Lagoon, Central Java) (Table 1). One specimen of *Stolephorusacinaces* Hata, Lavoué & Motomura, 2020 was selected as the outgroup.

**Table 1.** Taxonomic treatment and molecular marker sampling of 32 specimens of Stolephorus examined in the molecular section of the present study. “-” indicates corresponding sequence not determined. Bold accession numbers indicate sequences determined during the study. (Abbreviations: Cytb, cytochrome *b*; COI, cytochrome oxidase I; Gb, GenBank; “**”, holotype; “*”, paratype.

Species	Voucher	Origin	Cytb	COI
*S.eldorado* sp. nov.	KAUM–I. 94509*	Ha Long Bay, northern Vietnam	MH380318	**OM672421**
	KAUM–I. 94517**	Ha Long Bay, northern Vietnam	MH380319	**OM672422**
	KAUM–I. 94519*	Ha Long Bay, northern Vietnam	MH380320	**OM672423**
	KAUM–I. 94520*	Ha Long Bay, northern Vietnam	MH380321	**OM672424**
	KAUM–I. 94521*	Ha Long Bay, northern Vietnam	MH380322	**OM672425**
	KAUM–I. 113142*	off Dong-gang, Pingtung, Taiwan	MH380323	**OM672426**
	KAUM–I. 113143*	off Dong-gang, Pingtung, Taiwan	MH380324	**OM672427**
	KAUM–I. 113144*	off Dong-gang, Pingtung, Taiwan	MH380325	**OM672428**
	KAUM–I. 113145*	off Dong-gang, Pingtung, Taiwan	MH380326	**OM672417**
	KAUM–I. 113146*	off Dong-gang, Pingtung, Taiwan	MH380327	**OM672418**
	KAUM–I. 113147*	off Dong-gang, Pingtung, Taiwan	MH380328	**OM672419**
	KAUM–I. 113148*	off Dong-gang, Pingtung, Taiwan	MH380329	**OM672429**
	KAUM–I. 113149*	off Dong-gang, Pingtung, Taiwan	MH380330	**OM672420**
	KAUM–I. 113150*	off Dong-gang, Pingtung, Taiwan	MH380331	**OM672430**
	KAUM–I. 113151*	off Dong-gang, Pingtung, Taiwan	MH380332	**OM672431**
	NTUM12426 (Bgk15)	Bangkok, Thailand	MH380652	**OM672414**
	NTUM12426 (Bgk17)	Bangkok, Thailand	MH380653	**OM672415**
	- (Bgk39)	Bangkok, Thailand	MH380333	**OM672416**
	- (HK01)	Hong Kong	MH380334	**OM672413**
	20180725PZ25	Zhangzhou city, China (24.26N, 118.11°E) (Gb)	MH732976	MH732976
*S.diabolus* sp. nov.	IPMB-I 13.00001**	Telok Bahang, Penang Island, Malaysia	MT080882	MT080410
	- (larvae not preserved)	Klang Strait, West Peninsular Malaysia (Gb)	-	MH673948
*S.bengalensis* (Dutt & Babu Rao, 1959)	-	off Kochi, Kerala, India (9.97°N, 76.28°E) (Gb)	-	KU871055
	-	off Kochi, Kerala, India (9.97°N, 76.28°E) (Gb)	-	KU871061
	-	off Kochi, Kerala, India (9.97°N, 76.28°E) (Gb)	-	KU894592
	-	off Kochi, Kerala, India (9.97°N, 76.28°E) (Gb)	-	KU894597
	-	off Kochi, Kerala, India (9.97°N, 76.28°E) (Gb)	-	KU894598
	-	off Kochi, Kerala, India (9.97°N, 76.28°E) (Gb)	-	KU894599
	-	off Kochi, Kerala, India (9.97°N, 76.28°E) (Gb)	-	KU894600
	-	off Kochi, Kerala, India (9.97°N, 76.28°E) (Gb)	-	KU894601
*Stolephorus* sp.	- (larvae not preserved)	Segara Anakan lagoon, Central Java (Gb)	-	KY944580
**Outgroup**:				
*S.andhraensis* Babu Rao, 1966	NTUM12328 (Bg14)	Bangkok, Thailand	MH380656	MH380744

DNA was extracted from ethanol-preserved tissue samples. Polymerase chain reaction (PCR) amplification and sequencing of the COI gene followed standard protocols (Ward et al. 2005), with annealing at 55 °C. Amplification of the partial COI gene used the following primers: forward COI_FishF1 (5’-TCA ACC AAC CAC AAA GAC ATT GGC AC-3’) and reverse COI_FishR2 (5’-ACT TCA GGG TGA CCG AAG AAT CAG AA-3’) (Ward et al. 2005). PCR products were purified and sequenced in both directions by Sanger Sequencing technology using the same PCR primers. Sequences generated in this study have been deposited in the GenBank database (accession numbers given in Table 1).

Alignments of the cytochrome *b* and COI sequences were determined separately by eye, requiring neither insertions nor deletions. The final alignment combining the two genes (for 31 specimens plus one outgroup) comprised 1788 nucleotide positions. Uncorrected pairwise genetic distances (i.e. p-distances) amongst species were calculated with MEGA X (Stecher et al. 2020). The relationships between specimens were inferred by the Maximum Likelihood (ML) method of phylogenetic reconstruction using the general time-reversible model of nucleotide substitution with rate heterogeneity following a discrete gamma distribution (GTR + Г), using the software RAxML-NG (Kozlov et al. 2019) as implemented in the graphical interface raxmlGUI 2.0 (Edler et al. 2020). The tree was rooted using a specimen of *S.acinaces* and the robustness of each node determined by bootstrap support (500 replicates).

## ﻿Results and discussions

### ﻿ Stolephorus bengalensis

Taxon classificationAnimaliaClupeiformesEngraulidae

(Dutt & Babu Rao, 1959)

67E69026-8C76-5B86-84F8-C5E877CDE39C

**Figure 2.**
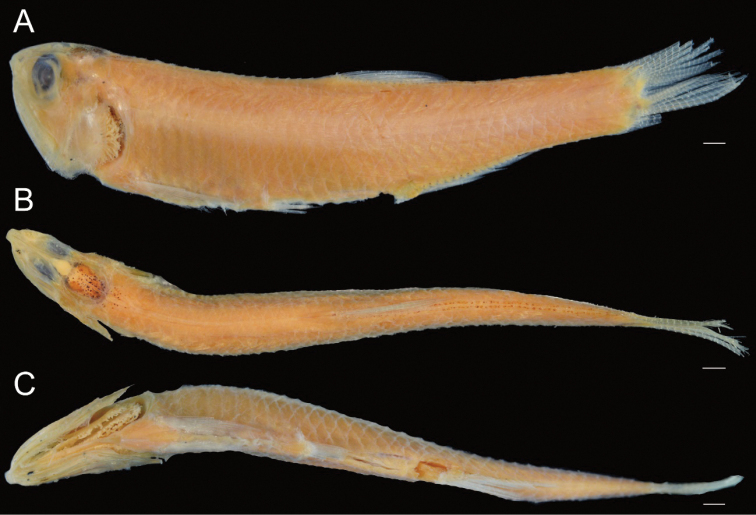
**A** lateral **B** dorsal and **C** ventral views of neotype of *Stolephorusbengalensis* (USNM 276476, 45.6 mm SL, Gulf of Mannar, India). Scale bars indicate 2 mm.

**Figure 3.**
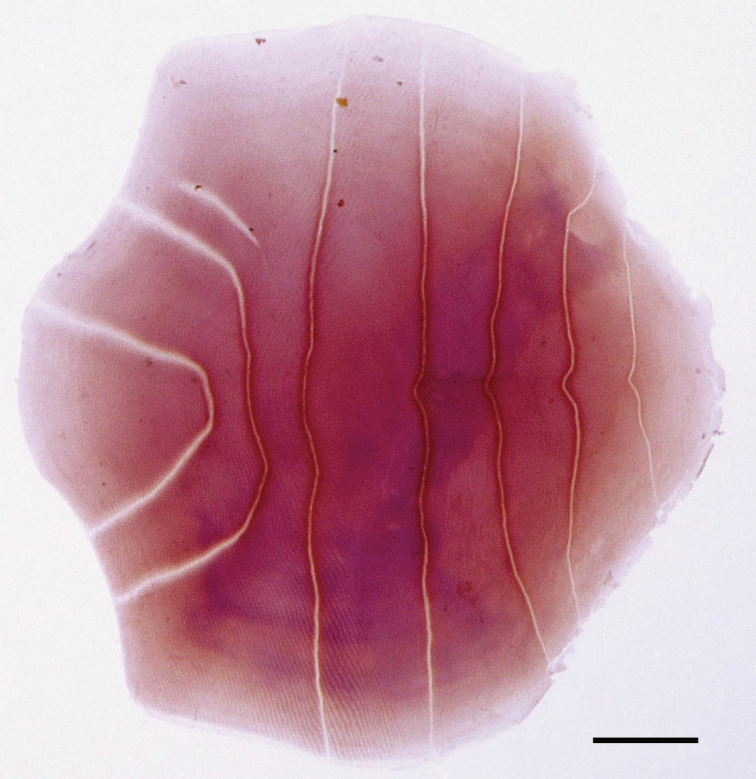
Stained scale removed from right side of mid-body (above anal fin) of *Stolephorusbengalensis*, BMNH 1969.5.30.34–45, 47.8 mm SL, Chennai, India (left-right inverted; scale bar indicates 0.5 mm).

[English name: Hardenberg’s Anchovy] Figs 2 , 3 ; Tables 2 , 3 , 4

Anchoviella baganensis bengalensis Dutt & Babu Rao, 1959: 160 [original locality: Waltair and Kakinada, Andhra Pradesh, east coast of India; type locality: Kilakarai, Gulf of Mannar, India, based on the neotype designated by Hata et al. (2019)].
Stolephorus baganensis macrops (lapsus memoriae for Stolephorusmegalops) (not of Delsman): Whitehead 1967 (in part): 18 (Bay of Bengal).
Stolephorus insularis (not of Delsman): Whitehead et al. 1988 (in part): 413 (northern part of Indian Ocean); Young et al. 1994: 222, fig. 7 (Wangkun and Fangliao, Taiwan); Wongratana et al. 1999 (in part): 1736 (northern part of Indian Ocean); Gangan et al. 2020: 562, fig. 5 (Kochi, Kerala State, India).
Stolephorus bengalensis : Hata et al. 2019 (in part): 24, fig. 12 (Pakistan and India; neotype designation).

#### Neotype.

USNM 276476, 45.8 mm SL, Kilakarai, Gulf of Mannar, India, 20 Feb 1964, J. W. Reintjes and P. S. B. R. James.

#### Non-type specimens.

46 specimens, 30.8–58.7 mm SL. **India**: BMNH 1969.5.30.34–45, 12 specimens, Chennai, Tamil Nadu State; OCF-P 10435, 4 specimens, 30.8–38.1 mm SL, estuary of Hooghly River, West Bengal State (purchased in fish market in Kolkata, West Bengal State); USNM 204227, 21 specimens, 42.7–51.8 mm SL, Sassan Docks, Mumbai, Maharashtra State. **Pakistan**: KAUM–I. 69286, 50.0 mm SL, KAUM–I. 69287, 58.7 mm SL, KAUM–I. 69288, 50.5 mm SL, KAUM–I. 69289, 54.4 mm SL, KAUM–I. 69290, 49.0 mm SL, KAUM–I. 69291, 53.1 mm SL, KAUM–I. 69292, 47.3 mm SL, KAUM–I. 69294, 58.6 mm, KAUM–I. 69295, 58.6 mm, West Wharf, Karachi.

#### Diagnosis.

A species of *Stolephorus* with the following combination of characters: 1UGR 16–19 (modally 18), 1LGR 23–27 (25), 1TGR 40–45 (44); 2UGR 11–15 (13), 2LGR 21–25 (23), 2TGR 33–39 (36); 3UGR 10–12 (11), 3LGR 13–15 (13), 3TGR 23–27 (24); 4UGR 7–9 (8), 4LGR 10–12 (11), 4TGR 17–21 (19); prepelvic scutes 5–7 (6); total vertebrae 40 or 41 (40); long maxilla, posterior tip just reaching or slightly short of posterior margin of opercle; predorsal scutes present; pelvic scute without spine; body scales deciduous; posterior border of pre-opercle concave, indented; paired dark patch on parietal area with little following pigmentation; distinct double pigment lines along dorsum posterior to dorsal fin; black spots below eye and on lower-jaw tip absent; anal-fin base long, 19.0–21.3% (20.2%) of SL; maximum orbit diameter 7.3–8.6% (8.1%) of SL; third dorsal-fin ray long, 18.5–19.9% (19.0%) of SL; pelvic fin rather long, 9.4–11.0% (10.2%) of SL, its posterior tip not reaching to vertical through dorsal-fin origin when depressed in specimens > 50 mm SL; distance between posterior ends of supramaxilla and maxilla 5.3–6.6% (5.8%) of SL.

#### Description.

Data for neotype presented first, followed by data for non-type specimens in parentheses (if different). Counts and measurements, expressed as percentages of SL or HL, given in Tables 2 and 3. Body laterally compressed, elongate, deepest at dorsal-fin origin. Dorsal profile of head and body slightly convex from snout tip to dorsal-fin origin, gently lowering to uppermost point of caudal-fin base. Ventral profile of head and body slightly convex from lower jaw tip to pelvic-fin insertion, thereafter, slowly rising to lowermost point of caudal-fin base. Single spine-like scute just anterior to dorsal-fin origin. Abdomen somewhat rounded, covered with seven (four to seven) spine-like prepelvic scutes. Pelvic scute without spine. Postpelvic scutes absent. Anus just anterior to anal-fin origin. Snout tip rounded; snout length less than eye diameter. Mouth large, inferior, ventral to body axis, extending backwards beyond posterior margin of eye. Maxilla long, its posterior tip pointed, just reaching (or slightly short of) opercle posterior margin. Lower jaw slender. Single row of conical teeth on both jaws and palatine. Patch of fine conical teeth on pterygoid. Several distinct conical teeth on vomer. Several rows of conical teeth on upper edges of basihyal and basibranchial. Eye large, round, covered with adipose eyelid, positioned laterally on head dorsal to horizontal through pectoral-fin insertion, visible in dorsal view. Pupil round. Orbit elliptical. Nostrils close to each other, anterior to orbit. Posterior margin of pre-opercle concave, indented. Subopercle and opercle with smoothly rounded posterior margins. Gill membrane without serrations. Interorbital space flat, width less than eye diameter. Pseudobranchial filaments present, length of longest filament less than eye diameter. Gill rakers long, slender, rough, visible from side of head when mouth opened. Single row of asperities on anterior surface of gill rakers. Isthmus muscle long, reaching anteriorly to posterior margin of gill membranes. Urohyal hidden by isthmus muscle, not visible without dissection. Gill membrane on each side joined distally, most of isthmus muscle exposed, not covered by gill membrane. Scales on lateral surface of body thin, cycloid, deciduous, those on lateral body surface with several centrally continuous vertical grooves and several longitudinal striae anteriorly (Fig. 3). Head scales absent. Fins scaleless, except for broad triangular sheath of scales on caudal fin. Dorsal-fin origin posterior to vertical through base of last pelvic-fin ray, slightly posterior to middle of body. Dorsal and anal fins with three anteriormost rays unbranched. First dorsal- and anal-fin rays minute. Anteriormost three rays of both dorsal and anal fins closely spaced. Anal-fin origin just below base of tenth (ninth to eleventh) dorsal-fin ray. Posterior tip of depressed anal fin not reaching caudal-fin base. Uppermost pectoral-fin ray unbranched, inserted below body axis. Posterior tip of pectoral fin not reaching to pelvic fin insertion. Dorsal, ventral and posterior margins of pectoral fin nearly linear. Pelvic fin shorter than pectoral fin, insertion anterior to vertical through dorsal-fin origin. Posterior tip of depressed pelvic fin not reaching to vertical through dorsal-fin origin. Caudal fin forked, posterior tips pointed.

**Table 2.** Meristics of specimens of *Stolephorusbengalensis* and *Stolephoruseldorado* sp. nov.

	*Stolephorusbengalensis*			*Stolephoruseldorado* sp. nov.		
	Neotype of *Anchoviellabaganensisbengalensis*	Non-types		Holotype	Paratypes	
	USNM 276476	*n* = 46		KAUM–I. 94517	*n* = 57	
Standard length (mm)	45.8	30.8–58.7	Modes ± SD	44.4	37.5–58.8	Modes ± SD
Dorsal-fin rays (unbranched)	3	3	3 ± 0	3	3	3 ± 0
Dorsal-fin rays (branched)	12	11–14	13 ± 0.7	13	11–14	13 ± 0.6
Anal-fin rays (unbranched)	3	3	3 ± 0	3	3*	3 ± 0.1
Anal-fin rays (branched)	18	16–20	18 ± 0.9	18	16–19	18 ± 0.6
Pectoral-fin rays (unbranched)	1	1	1 ± 0	1	1	1 ± 0
Pectoral-fin rays (branched)	11	10–12	11 ± 0.7	12	9–13	11 ± 0.8
Pelvic-fin rays (unbranched)	1	1	1 ± 0	1	1	1 ± 0
Pelvic-fin rays (branched)	6	6	6 ± 0	6	6	6 ± 0
Gill rakers on 1^st^ gill arch (upper)	16	17–19	18 ± 0.8	18	16–21	18 ± 1.1
Gill rakers on 1^st^ gill arch (lower)	24	23–27	25 ± 1.1	26	23–28	25 ± 1.0
Gill rakers on 1^st^ gill arch (total)	40	40–45	44 ± 1.5	44	40–47	42 ± 1.7
Gill rakers on 2^nd^ gill arch (upper)	13	11–15	13 ± 0.8	12	10–14	13 ± 0.8
Gill rakers on 2^nd^ gill arch (lower)	22	21–25	23 ± 0.9	24	20–24	23 ± 1.0
Gill rakers on 2^nd^ gill arch (total)	35	33–39	36 ± 1.5	36	30–38	36 ± 1.5
Gill rakers on 3^rd^ gill arch (upper)	10	10–12	11 ± 0.6	10	8–12	10 ± 0.8
Gill rakers on 3^rd^ gill arch (lower)	13	13–15	13 ± 0.6	13	12–14	13 ± 0.6
Gill rakers on 3^rd^ gill arch (total)	23	23–27	24 ± 1.1	23	20–26	23 ± 1.1
Gill rakers on 4^th^ gill arch (upper)	9	7–9	8 ± 0.6	8	7–10	8 ± 0.7
Gill rakers on 4^th^ gill arch (lower)	10	10–12	11 ± 0.5	11	9–12	11 ± 0.8
Gill rakers on 4^th^ gill arch (total)	19	17–21	19 ± 1.0	19	16–22	18 ± 1.3
Gill rakers on posterior face of 3^rd^ gill arch	6	4–7	5 ± 0.7	5	4–7	5 ± 0.7
Prepelvic scutes	7	5–7	6 ± 0.5	6	5–7	6 ± 0.6
Scale rows in longitudinal series	35	34–36	35 ± 0.7	34	34–36	35 ± 0.7
Transverse scales	8	8	8 ± 0	8	8–9	8 ± 0.3
Pseudobranchial filaments	broken	13–18	16 ± 1.3	17	14–18	16 ± 1.2
Total vertebrae	40	40–41	40 ± 0.4	39	38–40	39 ± 0.7

*one specimen with 4 unbranched anal-fin rays.

**Table 3.** Morphometrics of specimens of *Stolephorusbengalensis* and *Stolephoruseldorado* sp. nov.

	*Stolephorusbengalensis*			*Stolephoruseldorado* sp. nov.		
	Neotype of *Anchoviellabaganensisbengalensis*	Non-types		Holotype	Paratypes	
	USNM 276476	*n* = 46		KAUM–I. 94517	*n* = 57	
Standard length (mm)	45.8	30.8–58.7	Means ± SD	44.4	37.5–58.8	Means ± SD
As % SL						
Head length	25.8	23.0–26.1	24.7 ± 0.8	26.1	22.8–27.8	25.7 ± 1.3
Body depth	20.7	19.8–22.9	21.5 ± 0.7	17.9	17.3–22.0	20.3 ± 1.3
Pre-dorsal-fin length	56.8	52.3–57.1	54.5 ± 1.3	53.7	51.6–56.5	54.0 ± 1.2
Snout tip to pectoral-fin insertion	29.0	25.1–27.9	26.4 ± 0.8	28.0	22.5–29.2	26.9 ± 1.6
Snout tip to pelvic-fin insertion	47.6	42.4–49.4	45.1 ± 1.3	45.4	43.9–48.6	46.1 ± 1.2
Snout tip to anal-fin origin	66.3	61.3–66.5	64.2 ± 1.2	61.4	61.3–66.5	63.6 ± 1.2
Dorsal-fin base length	13.4	13.3–15.6	14.5 ± 0.6	14.3	13.2–15.7	14.6 ± 0.5
Anal-fin base length	20.8	19.0–21.3	20.2 ± 0.6	20.4	19.0–22.3	20.4 ± 0.8
Caudal-peduncle length	17.4	16.0–20.0	18.0 ± 1.1	18.1	16.4–19.8	18.2 ± 1.0
Caudal-peduncle depth	10.0	9.3–11.2	10.3 ± 0.4	9.3	8.7–10.9	9.7 ± 0.6
D–P1	37.2	33.9–38.1	35.9 ± 1.1	34.7	34.2–39.6	36.3 ± 1.2
D–P2	23.0	21.3–25.9	23.8 ± 1.0	20.4	19.1–26.1	23.2 ± 1.7
D–A	22.0	21.3–24.2	22.7 ± 0.8	20.2	19.2–23.2	21.6 ± 1.1
P1–P2	22.1	17.3–22.2	19.3 ± 1.4	19.6	16.9–23.8	20.3 ± 1.8
P2–A	19.7	15.9–20.3	18.6 ± 1.1	17.3	16.1–20.3	18.2 ± 1.0
Pectoral-fin length	broken	15.9–16.9	16.4 ± 0.4	18.0	14.9–18.5	16.5 ± 0.8
Pelvic-fin length	broken	9.4–11.0	10..2 ± 0.4	10.6	9.1–11.0	10.0 ± 0.5
Maxilla length	broken	19.7–22.3	21.0 ± 0.7	21.5	19.6–22.9	21.4 ± 0.9
Lower-jaw length	17.1	15.3–17.6	16.3 ± 0.5	17.3	14.6–17.9	16.7 ± 0.7
Supramaxilla end to maxilla end	broken	5.3–6.6	5.8 ± 0.3	5.1	5.0–6.3	5.6 ± 0.4
Maximum orbit diameter	8.5	7.3–8.6	8.1 ± 0.3	9.3	8.2–9.9	8.9 ± 0.4
Eye diameter	6.9	6.1–7.6	6.9 ± 0.3	8.0	6.4–8.6	7.5 ± 0.5
Snout length	4.4	3.4–4.0	3.7 ± 0.2	3.7	3.1–4.3	3.7 ± 0.3
Interorbital width	6.74	5.2–6.3	5.9 ± 0.3	5.8	4.9–6.2	5.8 ± 0.3
Postorbital length	12.8	11.8–14.1	13.0 ± 0.5	12.3	11.6–14.9	12.9 ± 0.7
1^st^ dorsal-fin ray length	0.9	0.9–2.1	1.5 ± 0.3	1.9	0.9–2.2	1.5 ± 0.3
2^nd^ dorsal-fin ray length	7.3	6.6–8.9	7.7 ± 0.6	broken	5.9–8.1	7.3 ± 0.6
3^rd^ dorsal-fin ray length	18.8	18.5–19.9	19.0 ± 0.4	broken	15.9–18.6	17.4 ± 0.8
1^st^ anal-fin ray length	1.9	1.0–2.0	1.6 ± 0.3	1.5	0.8–2.2	1.6 ± 0.3
2^nd^ anal-fin ray length	5.2	4.6–6.3	5.4 ± 0.5	6.5	4.1–6.5	5.2 ± 0.8
3^rd^ anal-fin ray length	13.0	14.0–16.5	15.0 ± 0.8	14.4	13.3–15.5	14.1 ± 1.8

Abbreviations: D–P1 (distance from dorsal-fin origin to pectoral-fin insertion); D–P2 (distance from dorsal-fin origin to pelvic-fin insertion); D–A (distance between origins of dorsal and anal fins); P1–P2 (distance between insertions of pectoral and pelvic fins); P2–A (distance between pelvic-fin insertion and anal-fin origin).

#### Colour of preserved specimens.

Body uniformly pale ivory. A pair of distinct dark patches on parietal region, with little pigmentation on occipital area. Double pigmented lines dorsally posterior to dorsal fin. A few melanophores scattered anteriorly on snout. No black spots below eye and on lower-jaw tip. Melanophores scattered along bases of dorsal and anal fins. All fins transparent, melanophores scattered along fin rays of caudal fin and anterior parts of dorsal and anal fins.

#### Distribution.

**Figure 4.**
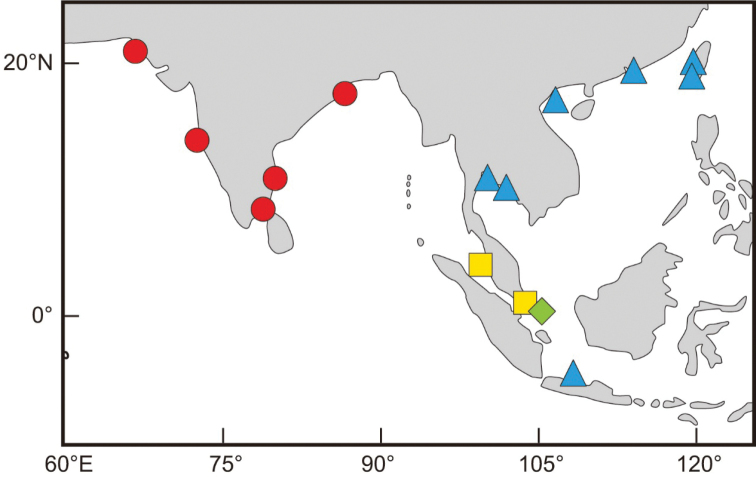
Map of the northern part of the Indo-West Pacific region showing distributional records of *Stolephorusbengalensis* (red circles), *S.diabolus* (yellow squares) sp. nov., *S.eclipsis* (green diamond) sp. nov. and *S.eldorado* (blue triangles) sp. nov., based on specimens examined in this study. Land masses outlined with black lines.

*Stolephorusbengalensis* is distributed in the northern Indian Ocean from Pakistan to the Bay of Bengal (Fig. 4). It is abundantly landed and marketed along the coast of the Bay of Bengal.

#### Morphological comparisons.

*Stolephorusbengalensis* has been considered conspecific with the three new species described herein, the four species being easily separable from all other congeners, except for *Stolephorusacinaces*, *Stolephorusandhraensis* Babu Rao, 1966, *Stolephoruscarpentariae* (De Vis, 1882), *Stolephorushindustanensis* Hata & Motomura, 2022, *Stolephorusholodon* (Boulenger, 1900), *Stolephorusronquilloi* Wongratana, 1983 and *Stolephorustamilensis* Gangan, Pavan-Kumar, Jahageerdar & Jaiswar, 2020, the former having a concavely indented pre-opercular margin and lacking a spine on the pelvic scute (Whitehead et al. 1988; Wongratana et al. 1999; Kimura et al. 2009; Hata and Motomura 2018a, b, c, d, e, 2021a, b, c, 2022; Hata et al. 2019, 2020a, b, 2021; Gangan et al. 2020). However, the former four species are distinguished from the other seven by having a predorsal scute (vs. absent in the latter) and double dark lines on the dorsum posterior to the dorsal fin (vs. no lines on the dorsum, except in *S.hindustanensis* and *S.ronquilloi*). Moreover, *S.carpentariae* also differs from *S.bengalensis* and the three new species in having 19 or 20 branched anal-fin rays [16–18 (rarely 19 or 20) in the remaining five species] and the anal-fin origin located below the origin of the second to sixth dorsal-fin ray (vs. eighth to eleventh) (Whitehead et al. 1988; Wongratana et al. 1999; Gangan et al. 2020; Hata et al. 2020b). *Stolephorusbengalensis*, *S.diabolus* sp. nov., *S.eclipsis* sp. nov. and *S.eldorado* sp. nov. resemble *Stolephorusbaganensis* Delsman, 1931, *Stolephorusdubiosus* Wongratana, 1983 and *Stolephorustri* (Bleeker, 1852) in having a predorsal scute and double pigment lines on the dorsum behind the dorsal fin, but differ in having deciduous body scales (vs. body scales not deciduous) and lacking a spine on the pelvic scute (pelvic scute with a hard posteriorly projecting spine) (Whitehead et al. 1988; Wongratana et al. 1999; Hata et al. 2019). Comparisons of *S.bengalensis* with *S.diabolus* sp. nov., *S.eclipsis* sp. nov. and *S.eldorado* sp. nov. are given in “Comparisons” under each new species.

#### Molecular comparisons.

**Figure 5.**
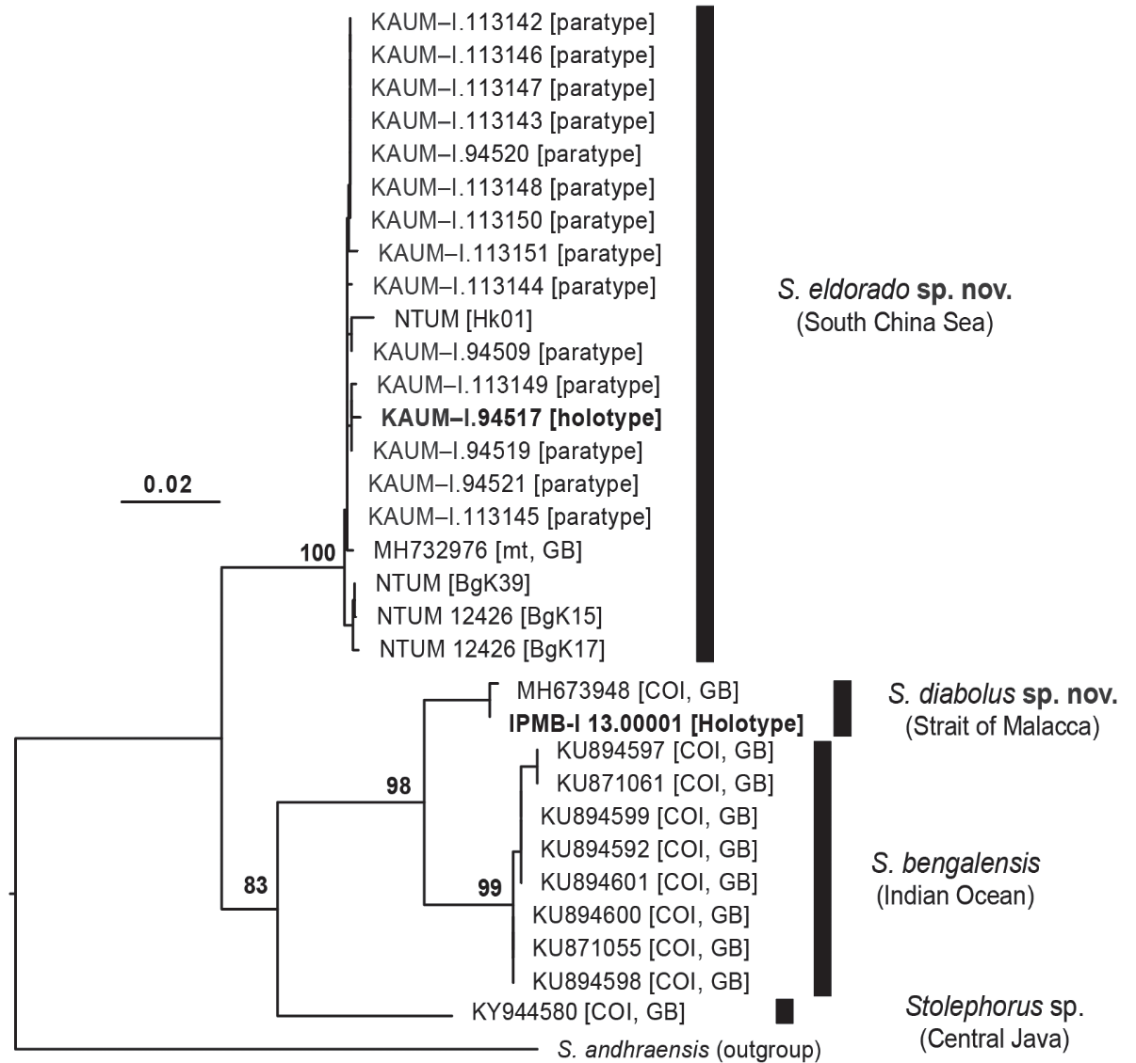
Maximum-likelihood phylogenetic tree of four species of *Stolephorus* related to *S.bengalensis*, based on the cytochrome *b* and cytochrome oxidase I genes (total: 1,788 base pairs) of 31 specimens, each species forming a monophyletic group. Each specimen identified by Museum Registration Number or GenBank number (see text and Table 1 for details). Type status (either holotype or paratype), specimen code, gene used (mt or COI) and sequence origin (GB) are indicated in brackets where necessary. Tree rooted by a specimen of *Stolephorusandhraensis*. Branch lengths proportional to number of substitutions. Bootstrap proportions indicated at nodes.

*Stolephorusbengalensis*, *S.diabolus* sp. nov. and *S.eldorado* sp. nov. were divergent from each other by at least 3.5% COI-based mean uncorrected genetic distance (min-max = 3.5–7.7%) (Fig. 5). In contrast, each species was genetically uniform, with intraspecific differentiation not exceeding 1% (note: *Stolephorus* sp. represented by a single specimen – see below), forming clear intraspecific *versus* interspecific genetic gaps. The ML phylogenetic tree using COI and cytochrome *b* markers (Fig. 5) was fully resolved, with interspecific relationships supported by bootstrap values above 80%. Each species formed a well-supported monophyletic group, in agreement with their genetic distinction, thereby confirming their taxonomic status, which was further supported by the morphological observations. The COI sequence of an unidentified larva collected from the Segara Anakan Lagoon, Central Java (Nuryanto et al. 2017), indicated either a range extension of *S.eclipsis* sp. nov. or the presence of an unidentified species of *Stolephorus*, related to *S.bengalensis*.

### ﻿ Stolephorus diabolus sp. nov.

Taxon classificationAnimaliaClupeiformesEngraulidae

89C301AA-78C9-595E-A68C-B5229FDCD7A8

https://zoobank.org/09515D76-5020-4C13-9391-7F325335C74C

**Figure 6.**
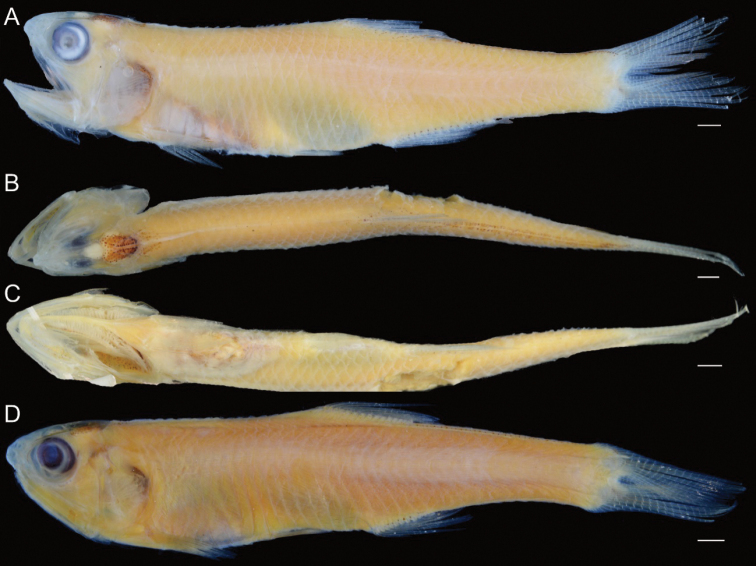
**A** lateral **B** dorsal and **C** ventral views of preserved holotype of *S.diabolus* sp. nov., IPMB-I 13.00001, 49.7 mm SL, Teluk Bahang, Penang, Malaysia **D** lateral view of preserved paratype of *S.diabolus* sp. nov., ZUMT 62056, 37.3 mm SL, Singapore. Scale bars: 2 mm.

[New English name: Demon Anchovy] Figs 1C , 6 ; Tables 4 , 5 , 6

Stolephorus bengalensis (not of Dutt and Babu Rao): Hata et al. 2022: (in part) 34 (Singapore).

#### Holotype.

IPMB-I 13.00001, 49.7 mm SL, Teluk Bahang, Penang, Malaysia.

#### Paratypes.

14 specimens, 28.5–43.7 mm SL. USMFC 82-0017, 43.7 mm SL, collected with the holotype; USMFC 82-0057, 4 specimens, 40.1–41.1 mm SL, estuary of Merbok River, Jeti Semeling, Malaysia; ZUMT 62056, 5 specimens, 28.5–38.4 mm SL, KAUM–I. 163702, 36.3 mm SL, KAUM–I. 163703, 36.4 mm SL, NSMT-P 143554, 36.4 mm SL, NSMT-P 143555, 36.6 mm SL, Singapore.

#### Diagnosis.

A species of *Stolephorus* with the following combination of characters: 1UGR 14–16 (modally 16), 1LGR 20–23 (22), 1TGR 35–38 (38); 2UGR 10 or 11 (11), 2LGR 19 or 20 (20), 2TGR 30 or 31 (31); 3UGR 8 or 9 (9), 3LGR 11 or 12 (12), 3TGR 20 or 21 (21); 4UGR 6 or 7 (7), 4LGR 9 or 10 (9), 4TGR 15–17 (17); prepelvic scutes 5–7 (6); total vertebrae 39; long maxilla, posterior tip just reaching or slightly short of posterior margin of opercle; predorsal scute present; pelvic scute without spine; body scales deciduous; posterior border of pre-opercle concave, indented; paired dark patch on parietal area with little following pigmentation; distinct double pigment lines along dorsum posterior to dorsal fin; black spots below eye and on lower-jaw tip absent; anal-fin base long, 19.8–22.3% (mean 20.7%) of SL; maximum orbit diameter 8.1–8.7% (8.3%) of SL; third dorsal-fin ray short, 17.0–18.5% (18.0%) of SL; pelvic fin rather long, 9.6–11.3% (10.0%) of SL, its posterior tip not reaching to vertical through dorsal-fin origin when depressed in specimens > 40 mm SL; distance between posterior ends of supramaxilla and maxilla 5.7–6.4% (6.1%) of SL.

**Table 4.** Frequency distribution of total vertebral numbers of *Stolephorusbengalensis*, *Stolephorusdiabolus* sp. nov., *Stolephoruseclipsis* sp. nov. and *Stolephoruseldorado* sp. nov.

	Total vertebrae				
		38	39	40	41
*Stolephorusbengalensis*	*n* = 32			27	5
*Stolephorusdiabolus* sp. nov.	*n* = 2		2		
*Stolephoruseclipsis* sp. nov.	*n* = 14	6	8		
*Stolephoruseldorado* sp. nov.	*n* = 45	11	24	10	

#### Description.

Data for holotype presented first, followed by data for paratypes in parentheses (if different). Counts and measurements, expressed as percentages of SL or HL, given in Tables 5 and 6. Body laterally compressed, elongate, deepest at dorsal-fin origin. Dorsal profile of head and body slightly convex from snout tip to dorsal-fin origin, gently lowering to uppermost point of caudal-fin base. Ventral profile of head and body slightly convex from lower jaw tip to pelvic-fin insertion, thereafter, slowly rising to lowermost point of caudal-fin base. Single spine-like scute just anterior to dorsal-fin origin. Abdomen somewhat rounded. Scutes on ventrum broken in holotype (five to seven spine-like prepelvic scutes on ventrum in paratypes). Pelvic scute without spine. Postpelvic scutes absent. Anus just anterior to anal-fin origin. Snout tip rounded; snout length less than eye diameter. Mouth large, inferior, ventral to body axis, extending backwards beyond posterior margin of eye. Maxilla long, its posterior tip broken in holotype (posterior pointed, just reaching or slightly short of opercle posterior margin in paratypes). Lower jaw slender. Single row of conical teeth on both jaws and palatine. Patch of fine conical teeth on pterygoid. Several distinct conical teeth on vomer. Several rows of conical teeth on upper edges of basihyal and basibranchial. Eye large, round, covered with adipose eyelid, positioned laterally on head dorsal to horizontal through pectoral-fin insertion, visible in dorsal view. Pupil round. Orbit elliptical. Nostrils close to each other, anterior to orbit. Posterior margin of pre-opercle concave, indented. Subopercle and opercle with smoothly rounded posterior margins. Gill membrane without serrations. Interorbital space flat, width less than eye diameter. Pseudobranchial filaments present, length of longest filament less than eye diameter. Gill rakers long, slender, rough, visible from side of head when mouth opened. Single row of asperities on anterior surface of gill rakers. Isthmus muscle long, reaching anteriorly to posterior margin of gill membranes. Urohyal hidden by isthmus muscle, not visible without dissection. Gill membrane on each side joined distally, most of isthmus muscle exposed, not covered by gill membrane. Body scales deciduous, completely lacking on specimens, except for prepelvic scutes. Head scales absent. Fins scaleless, except for broad triangular sheath of scales on caudal fin. Dorsal-fin origin posterior to vertical through base of last pelvic-fin ray, slightly posterior to middle of body. Dorsal and anal fins with three anteriormost rays unbranched. First dorsal- and anal-fin rays minute. Anteriormost three rays of both dorsal and anal fins closely spaced. Anal-fin origin just below base of eighth (eighth to eleventh) dorsal-fin ray. Posterior tip of depressed anal fin not reaching caudal-fin base. Uppermost pectoral-fin ray unbranched, inserted below body axis. Posterior tip of pectoral fin not reaching to pelvic fin insertion. Dorsal, ventral and posterior margins of pectoral fin nearly linear. Pelvic fin shorter than pectoral fin, insertion anterior to vertical through dorsal-fin origin. Posterior tip of depressed pelvic fin not reaching to vertical through dorsal-fin origin (reaching to vertical through first to sixth dorsal-fin ray origin in some paratypes smaller than 40 mm SL). Caudal fin forked, posterior tips pointed.

**Table 5.** Meristics of specimens of *Stolephorusdiabolus* sp. nov. and *Stolephoruseclipsis* sp. nov.

	*Stolephorusdiabolus* sp. nov.			*Stolephoruseclipsis* sp. nov.		
	Holotype	Paratypes		Holotype	Paratypes	
	IPMB-I 13.00001	*n* = 14		MZB 26452	*n* = 28	
Standard length (mm)	49.7	28.5–43.7	Modes ± SD	40.3	32.0–43.7	Modes ± SD
Dorsal-fin rays (unbranched)	3	3	3 ± 0	3	3	3 ± 0
Dorsal-fin rays (branched)	12	12–13	13 ± 0.5	12	11–13	12 ± 0.4
Anal-fin rays (unbranched)	3	3	3 ± 0	3	3	3 ± 0
Anal-fin rays (branched)	16	16–18	17 ± 0.7	17	16–18	17 ± 0.7
Pectoral-fin rays (unbranched)	1	1	1 ± 0	1	1	1 ± 0
Pectoral-fin rays (branched)	11	10–13	11 ± 0.8	10	10–12	11 ± 0.6
Pelvic-fin rays (unbranched)	1	1	1 ± 0	1	1	1 ± 0
Pelvic-fin rays (branched)	6	6	6 ± 0	6	6	6 ± 0
Gill rakers on 1^st^ gill arch (upper)	16	14–16	16 ± 0.6	20	19–21	20 ± 0.7
Gill rakers on 1^st^ gill arch (lower)	22	20–23	22 ± 0.7	28	26–30	28 ± 0.8
Gill rakers on 1^st^ gill arch (total)	38	35–38	38 ± 1.0	48	47–51	47 ± 1.1
Gill rakers on 2^nd^ gill arch (upper)	10	11	11 ± 0.2	13	13–16	14 ± 0.7
Gill rakers on 2^nd^ gill arch (lower)	20	19–20	20 ± 0.4	25	24–27	25 ± 0.8
Gill rakers on 2^nd^ gill arch (total)	30	30–31	31 ± 0.4	38	37–42	39 ± 1.4
Gill rakers on 3^rd^ gill arch (upper)	9	8–9	9 ± 0.2	11	10–13	12 ± 0.7
Gill rakers on 3^rd^ gill arch (lower)	12	11–12	12 ± 0.5	14	14–16	15 ± 0.6
Gill rakers on 3^rd^ gill arch (total)	21	20–21	21 ± 0.5	25	25–28	27 ± 1.1
Gill rakers on 4^th^ gill arch (upper)	7	6–7	7 ± 0.4	8	8–11	9 ± 0.9
Gill rakers on 4^th^ gill arch (lower)	9	9–10	9 ± 0.5	11	11–13	12 ± 0.5
Gill rakers on 4^th^ gill arch (total)	16	15–17	17 ± 0.8	19	19–24	21 ± 1.2
Gill rakers on posterior face of 3^rd^ gill arch	3	3–5	4 ± 0.5	4	4–7	5 ± 0.7
Prepelvic scutes	broken	5–7	6 ± 0.5	6	5–7	6 ± 0.5
Scale rows in longitudinal series	34	34–35	35 ± 0.5	35	35–36	35 ± 0.4
Transverse scales	8	8–9	8 ± 0.2	8	8	8 ± 0
Pseudobranchial filaments	broken	14–16	15 ± 0.7	14	14–18	15 ± 1.2
Total vertebrae	39	39	39 ± 0	38	38–39	39 ± 0.5

**Table 6.** Morphometrics of specimens of *Stolephorusdiabolus* sp. nov. and *Stolephoruseclipsis* sp. nov.

	*Stolephorusdiabolus* sp. nov.			*Stolephoruseclipsis* sp. nov.		
	Holotype	Paratypes		Holotype	Paratypes	
	IPMB-I 13.00001	*n* = 14		MZB 26452	*n* = 28	
Standard length (mm)	49.7	28.5–43.7	Means ± SD	40.3	32.0–43.7	Means ± SD
As % SL						
Head length	24.8	25.0–25.9	25.4 ± 0.3	25.4	23.6–26.7	24.8 ± 0.8
Body depth	21.7	19.8–21.9	20.9 ± 0.7	20.6	18.4–20.8	19.6 ± 0.6
Pre-dorsal-fin length	51.8	51.3–52.9	52.1 ± 0.5	52.8	51.3–54.9	53.4 ± 1.0
Snout tip to pectoral-fin insertion	25.7	26.2–28.4	27.2 ± 0.6	26.8	24.8–28.5	26.5 ± 0.9
Snout tip to pelvic-fin insertion	48.2	45.8–49.0	47.2 ± 0.8	47.0	44.8–47.3	46.2 ± 0.7
Snout tip to anal-fin origin	65.4	63.0–66.0	64.4 ± 0.9	63.1	62.8–65.8	64.1 ± 0.9
Dorsal-fin base length	15.0	13.9–16.6	15.0 ± 0.7	13.6	13.1–14.5	13.8 ± 0.4
Anal-fin base length	19.8	19.9–22.3	20.7 ± 0.8	19.3	17.6–19.3	18.6 ± 0.5
Caudal-peduncle length	18.8	16.4–19.4	17.8 ± 0.9	17.4	14.7–18.5	17.1 ± 0.9
Caudal-peduncle depth	9.8	9.4–10.3	9.8 ± 0.2	9.9	9.2–10.6	9.8 ± 0.4
D–P1	36.5	33.8–36.4	35.3 ± 0.8	38.0	34.0–38.8	36.5 ± 1.3
D–P2	23.5	21.9–24.5	23.4 ± 0.6	24.1	21.0–23.9	22.5 ± 0.8
D–A	22.6	20.7–23.1	22.2 ± 0.8	21.3	20.0–21.9	20.9 ± 0.5
P1–P2	24.1	19.4–20.0	21.0 ± 1.1	21.3	19.3–22.8	21.0 ± 0.9
P2–A	18.0	16.3–19.5	18.0 ± 0.9	18.5	17.5–20.6	19.0 ± 0.8
Pectoral-fin length	16.2	15.4–17.1	16.2 ± 0.5	16.4	15.5–17.7	16.7 ± 0.6
Pelvic-fin length	9.8	9.6–11.3	10.0 ± 0.4	8.7	8.8–9.9	9.4 ± 0.3
Maxilla length	broken	20.9–21.9	21.4 ± 0.3	20.7	19.9–22.5	21.1 ± 0.7
Lower-jaw length	16.2	16.2–17.5	16.6 ± 0.3	16.4	15.8–17.7	16.6 ± 0.5
Supramaxilla end to maxilla end	broken	5.7–6.4	6.1 ± 0.2	5.2	4.7–5.4	5.1 ± 0.2
Maximum orbit diameter	8.2	8.1–8.7	8.3 ± 0.2	8.8	7.9–9.6	8.7 ± 0.4
Eye diameter	6.5	6.1–7.7	6.9 ± 0.5	6.8	6.7–8.3	7.3 ± 0.4
Snout length	3.7	3.6–4.2	3.8 ± 0.2	4.0	3.4–4.2	3.8 ± 0.2
Interorbital width	5.6	5.5–5.9	5.7 ± 0.2	5.7	5.5–6.2	5.8 ± 0.2
Postorbital length	12.8	12.9–14.2	13.4 ± 0.4	12.1	11.5–12.9	12.1 ± 0.4
1^st^ dorsal-fin ray length	1.5	0.8–2.2	1.6 ± 0.4	1.5	1.0–2.2	1.5 ± 0.3
2^nd^ dorsal-fin ray length	broken	7.0–9.8	8.1 ± 0.7	broken	5.1–5.7	7.6 ± 0.6
3^rd^ dorsal-fin ray length	broken	17.0–18.5	18.0 ± 0.5	17.1	16.5–18.8	17.6 ± 0.7
1^st^ anal-fin ray length	1.6	0.9–2.2	1.5 ± 0.4	1.9	1.2–2.2	1.7 ± 0.3
2^nd^ anal-fin ray length	5.3	4.8–7.2	5.5 ± 0.6	5.2	5.1–5.7	5.3 ± 0.2
3^rd^ anal-fin ray length	broken	14.6–16.0	15.4 ± 0.4	14.4	13.4–15.0	14.2 ± 0.5

Abbreviations: D–P1 (distance from dorsal-fin origin to pectoral-fin insertion); D–P2 (distance from dorsal-fin origin to pelvic-fin insertion); D–A (distance between origins of dorsal and anal fins); P1–P2 (distance between insertions of pectoral and pelvic fins); P2–A (distance between pelvic-fin insertion and anal-fin origin).

#### Colour of preserved specimens.

Body uniformly pale white. A pair of distinct dark patches on parietal region, with little pigmentation on occipital area. No black spots below eye and on lower-jaw tip. Melanophores scattered on posterior margins of scale pockets on dorsum. Double pigmented lines dorsally posterior to dorsal fin. Melanophores scattered along bases of dorsal and anal fins. All fins transparent, melanophores scattered along fin rays of caudal fin and anterior parts of dorsal and anal fins.

#### Distribution.

*Stolephorusdiabolus* sp. nov. is currently known only from the western coast of the Peninsular Malaysia (Merbok River Estuary and Penang) and Singapore (Fig. 4).

#### Etymology.

The specific name “*diabolus*” is derived from Latin meaning “demon”, in reference to the hard spine on the dorsum of the species.

#### Comparisons.

**Figure 7.**
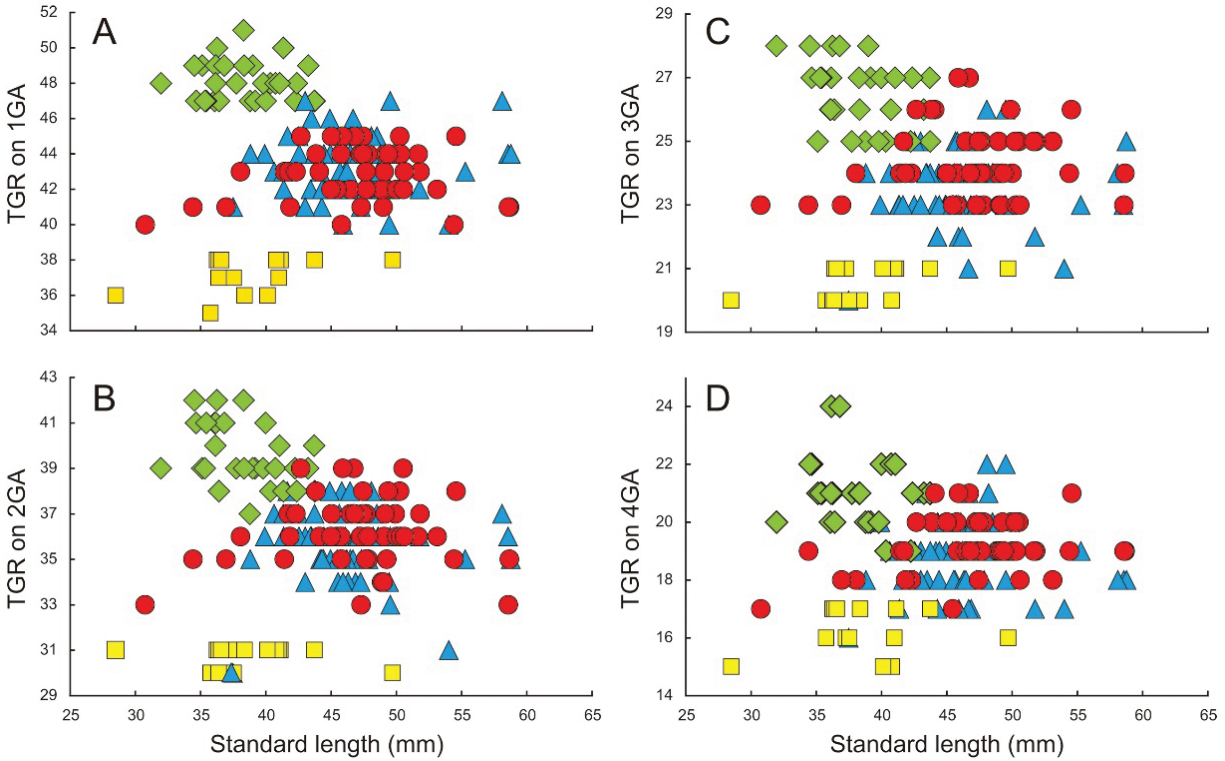
Relationships of total gill raker numbers (TGR) on **A** first gill arch (1GA) **B** second gill arch (2GA) **C** third gill arch (3GA) and **D** fourth gill arch (4GA) to standard length in *Stolephorusbengalensis* (red circles), *S.diabolus* sp. nov. (yellow squares), *S.eclipsis* sp. nov. (green diamonds) and *S.eldorado* sp. nov. (blue triangles).

**Figure 8.**
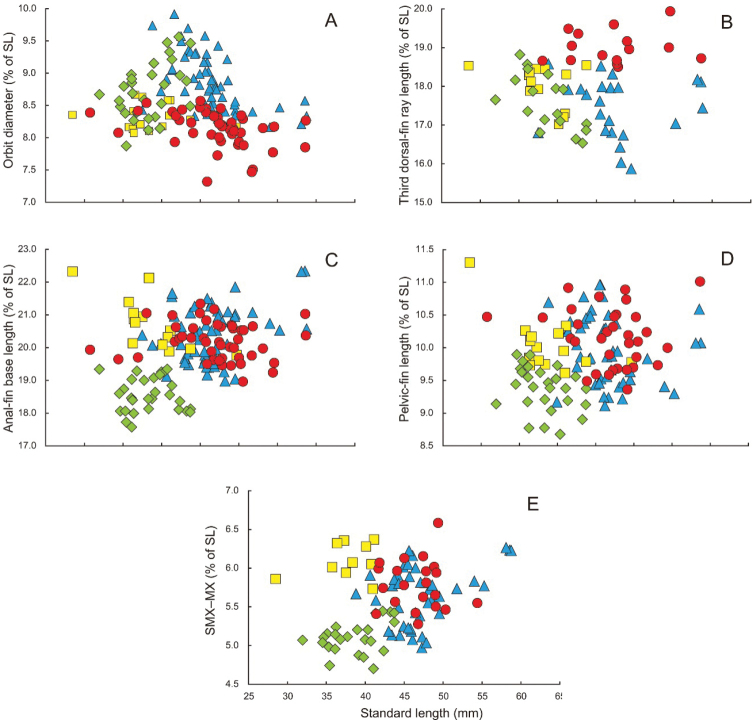
Relationships of **A** orbit diameter (as % of standard length; SL) **B** third dorsal-fin ray length (as % of SL) **C** anal-fin base length (as % of SL) **D** pelvic-fin length (as % of SL) and **E** distance between posterior ends of supramaxilla and maxilla (SMX–MX) in *Stolephorusbengalensis* (red circles), *S.diabolus* sp. nov. (yellow squares), *S.eclipsis* sp. nov. (green diamonds) and *S.eldorado* sp. nov. (blue triangles).

The new species is distinguished from *S.bengalensis*, *S.eclipsis* and *S.eldorado* by lower gill raker counts: 1TGR, 35–38 in *S.diabolus* (vs. 40 or more in the other three species); 2TGR, 30 or 31 in *S.diabolus* [vs. 33 or more (rarely 30 or 31 in *S.eldorado*)]; 3TGR, 20 or 21 in *S.diabolus* [vs. 22 or more in the other three species (rarely 21 in *S.eldorado*)]; and 4TGR, 15–17 in *S.diabolus* (vs. 17 or more) (Fig. 7). Moreover, *S.diabolus* has a shorter orbit diameter than *S.eldorado* [maximum orbit diameter 8.1–8.7% (mean 8.3%) of SL in *S.diabolus* vs. 8.2–9.9% (8.9%) in *S.eldorado*; Fig. 8A]. Furthermore, *S.diabolus* is distinguished from *S.bengalensis* by having a shorter third dorsal-fin ray [17.0–18.5% (mean 18.0%) of SL in *S.diabolus* vs. 18.5–19.9% (19.0%) in *S.bengalensis* (Fig. 8B)] and lower total vertebral numbers [39 vs. 40 or 41 (modally 40) (Table 4)]. Detailed comparisons of *S.diabolus* with *S.eclipsis* and *S.eldorado* are given in “Comparisons” under each species.

### ﻿ Stolephorus eclipsis sp. nov.

Taxon classificationAnimaliaClupeiformesEngraulidae

B61FE9B1-A485-59B9-9DA1-3C8F757769B6

https://zoobank.org/1556E6AA-0531-4361-874E-3FE6DB1FEA10

**Figure 9.**
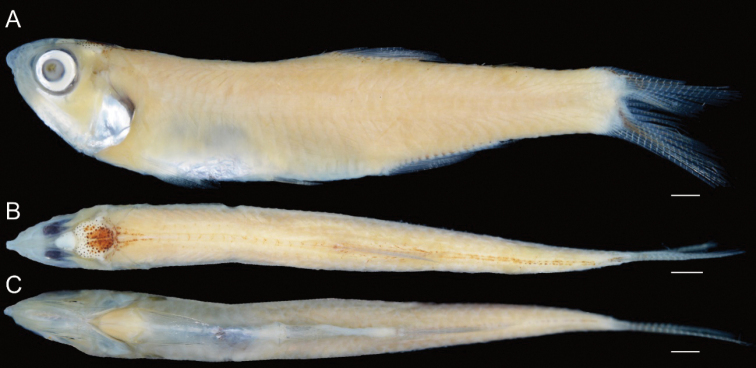
**A** lateral **B** dorsal and **C** ventral views of preserved holotype of *S.eclipsis* sp. nov., MZB 26452, 40.3 mm SL, Bintan Island, Riau Archipelago, Indonesia. Scale bars indicate 2 mm.

[New English name: Eclipse Anchovy] Fig. 9 ; Tables 4 , 5 , 6

#### Holotype.

MZB 26452, 40.3 mm SL, Bintan Island, Riau Archipelago, Indonesia.

#### Paratypes.

28 specimens, 32.0–43.7 mm SL. LBRC-F 5039, 35.4 mm SL, LBRC-F 5040, 35.3 mm SL, LBRC-F 5041, 36.1 mm SL, Tanjungpinang, Bintan Island, Riau Archipelago, Indonesia; MZB 26440, 32.0 mm SL, MZB 26441, 36.1 mm SL, MZB 26442, 35.1 mm SL, MZB 26443, 34.7 mm SL, MZB 26444, 34.5 mm SL, MZB 26445, 36.3 mm SL, MZB 26446, 36.2 mm SL, MZB 26447, 38.8 mm SL, MZB 26448, 39.2 mm SL, MZB 26449, 37.7 mm SL, MZB 26450, 40.0 mm SL, MZB 26451, 36.8 mm SL, 26453, 36.4 mm SL, MZB 26454, 39.0 mm SL, MZB 26455, 41.3 mm SL, MZB 26456, 43.7 mm SL, MZB 26457, 39.8 mm SL, MZB 26458, 40.7 mm SL, MZB 26459, 43.2 mm SL, MZB 26460, 43.7 mm SL, MZB 26461, 5 specimens, 38.3–42.4 mm SL, collected with the holotype.

#### Diagnosis.

A species of *Stolephorus* with the following combination of characters: 1UGR 19–21 (modally 20), 1LGR 26–30 (28), 1TGR 47–51 (47); 2UGR 13–16 (14), 2LGR 24–27 (25), 2TGR 37–42 (39); 3UGR 10–13 (12), 3LGR 14–16 (15), 3TGR 25–28 (27); 4UGR 8–11 (9), 4LGR 11–13 (12), 4TGR 19–24 (21); prepelvic scutes 5–7 (6); total vertebrae 38–39 (39); long maxilla, posterior tip just reaching or slightly short of posterior margin of opercle; predorsal scute present; pelvic scute without spine; body scales deciduous; posterior border of pre-opercle concave, indented; paired dark patch on parietal area with little following pigmentation; distinct double pigment lines along dorsum posterior to dorsal fin; black spots below eye and on lower-jaw tip absent; anal-fin base short, 17.6–19.3% (mean 18.6%) of SL; third dorsal-fin ray 16.5–18.8% (17.6%) of SL; pelvic fin short, 8.7–9.9% (9.4%) of SL, its posterior tip usually not reaching to vertical through dorsal-fin origin when depressed; distance between posterior ends of supramaxilla and maxilla 4.7–5.4% (5.1%) of SL; pre-dorsal-fin length 51.3–54.9% (53.4%) of SL; dorsal-fin base short, 13.1–14.5% (13.8%) of SL.

#### Description.

Data for holotype presented first, followed by data for paratypes in parentheses (if different). Counts and measurements, expressed as percentages of SL or HL, given in Tables 5 and 6. Body laterally compressed, elongate, deepest at dorsal-fin origin. Dorsal profile of head and body slightly convex from snout tip to dorsal-fin origin, gently lowering to uppermost point of caudal-fin base. Ventral profile of head and body slightly convex from lower jaw tip to pelvic-fin insertion, thereafter, slowly rising to lowermost point of caudal-fin base. Single spine-like scute just anterior to dorsal-fin origin. Abdomen somewhat rounded, covered with six (five to seven) spine-like prepelvic scutes. Pelvic scute without spine. Postpelvic scutes absent. Anus just anterior to anal-fin origin. Snout tip rounded; snout length less than eye diameter. Mouth large, inferior, ventral to body axis, extending backwards beyond posterior margin of eye. Maxilla long, its posterior tip pointed, just reaching (or slightly short of) opercle posterior margin. Lower jaw slender. Single row of conical teeth on both jaws and palatine. Patch of fine conical teeth on pterygoid. Several distinct conical teeth on vomer. Several rows of conical teeth on upper edges of basihyal and basibranchial. Eye large, round, covered with adipose eyelid, positioned laterally on head dorsal to horizontal through pectoral-fin insertion, visible in dorsal view. Pupil round. Orbit elliptical. Nostrils close to each other, anterior to orbit. Posterior margin of pre-opercle concave, indented. Subopercle and opercle with smoothly rounded posterior margins. Gill membrane without serrations. Interorbital space flat, width less than eye diameter. Pseudobranchial filaments present, length of longest filament less than eye diameter. Gill rakers long, slender, rough, visible from side of head when mouth opened. Single row of asperities on anterior surface of gill rakers. Isthmus muscle long, reaching anteriorly to posterior margin of gill membranes. Urohyal hidden by isthmus muscle, not visible without dissection. Gill membrane on each side joined distally, most of isthmus muscle exposed, not covered by gill membrane. Body scales deciduous, completely lacking on specimens, except for prepelvic scutes. Head scales absent. Fins scaleless, except for broad triangular sheath of scales on caudal fin. Dorsal-fin origin posterior to vertical through base of last pelvic-fin ray, slightly posterior to middle of body. Dorsal and anal fins with three anteriormost rays unbranched. First dorsal- and anal-fin rays minute. Anteriormost three rays of both dorsal and anal fins closely-spaced. Anal-fin origin just below base of eighth (eighth to eleventh) dorsal-fin ray. Posterior tip of depressed anal fin not reaching caudal-fin base. Uppermost pectoral-fin ray unbranched, inserted below body axis. Posterior tip of pectoral fin not reaching to pelvic fin insertion. Dorsal, ventral and posterior margins of pectoral fin nearly linear. Pelvic fin shorter than pectoral fin, insertion anterior to vertical through dorsal-fin origin. Posterior tip of depressed pelvic fin not reaching to vertical through dorsal-fin origin (reaching to vertical through first to third dorsal-fin ray origin in some paratypes). Caudal fin forked, posterior tips pointed.

#### Colour of preserved specimens.

Body uniformly pale ivory. A pair of distinct dark patches on parietal region, with little pigmentation on occipital area. Double pigmented lines dorsally posterior to dorsal fin. A few melanophores scattered anteriorly on snout. No black spots below eye and on lower-jaw tip. Melanophores scattered along bases of dorsal and anal fins. All fins transparent, melanophores scattered along fin rays of caudal fin and anterior parts of dorsal and anal fins.

#### Distribution.

*Stolephoruseclipsis* sp. nov. is currently known only from Bintan Island, Riau Archipelago, Indonesia (Fig. 4).

#### Etymology.

The specific name “*eclipsis*” refers to eclipse, reminiscent of the concave pre-opercle of the new species.

#### Comparisons.

**Figure 10.**
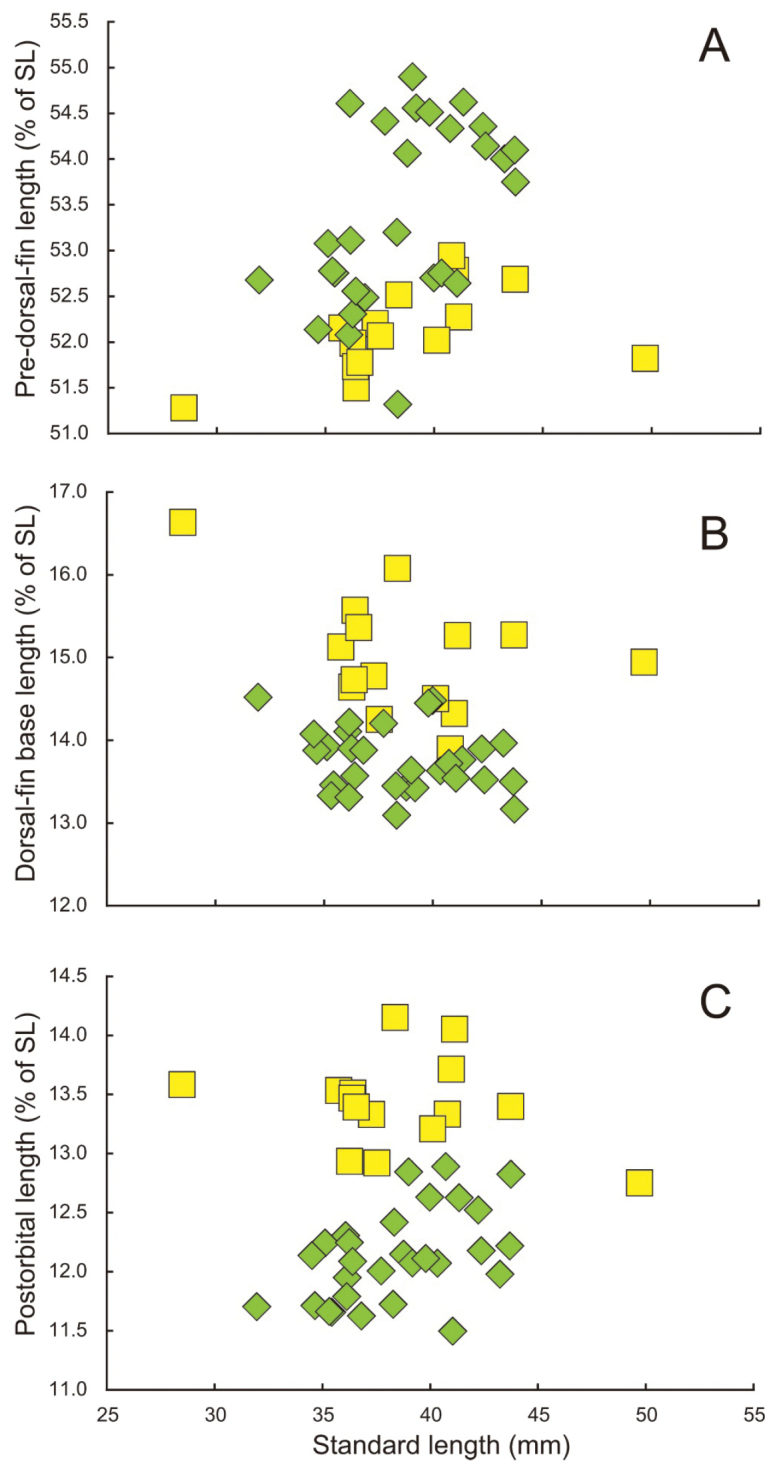
Relationships of **A** pre-dorsal-fin length (as % of standard length; SL) **B** dorsal-fin base length (as % of SL), and **C** postorbital length (as % of SL) in *Stolephorusdiabolus* sp. nov. (yellow squares) and *S.eclipsis* sp. nov. (green diamonds).

The new species differs from *S.bengalensis*, *S.diabolus* and *S.eldorado* in having higher gill raker counts [1TGR, 47–51 or more in *S.eclipsis* (vs. 47 or fewer in the other three species); 2TGR, 37–42 in *S.eclipsis* (vs. 39 or fewer); 3TGR, 25–28 in *S.eclipsis* (vs. 27 or fewer); and 4TGR, 19–24 in *S.eclipsis* (vs. 22 or fewer) (Fig. 7)], a shorter anal-fin base (17.6–19.3% of SL in *S.eclipsis* vs. 19.0–21.3% in *S.bengalensis*, 19.8–22.3% in *S.diabolus* and 19.0–22.3% in *S.eldorado*; Fig. 8C) and pelvic fin [8.7–9.9% (mean 9.4%) of SL in *S.eclipsis* vs. 9.4–11.0% (10.2%) in *S.bengalensis*, 9.6–11.3% (10.0%) in *S.diabolus* and 9.1–11.0% (10.0%) in *S.eldorado*; Fig. 8D] and shorter distance between the posterior ends of the supramaxilla and maxilla [4.7–5.4% (5.1%) of SL in *S.eclipsis* vs. 5.3–6.6% (5.8%) in *S.bengalensis*, 5.7–6.4% (6.1%) in *S.diabolus* and 5.0–6.3% (5.6%) in *S.eldorado*; Fig. 8E]. *Stolephoruseclipsis* also differs from *S.bengalensis* in having a shorter third dorsal-fin ray (16.5–18.8% of SL in *S.eclipsis* vs. 18.5–19.9% in *S.bengalensis*; Fig. 8B) and lower total vertebral number [38–39 (modally 39) vs. 40 or 41 (40) (Table 4)]. Moreover, *S.eclipsis* is distinguished from *S.diabolus* by a greater pre-dorsal-fin distance [51.3–54.9% (mean 53.4%) of SL in *S.eclipsis* vs. 51.3–52.9% (52.1%) in *S.diabolus*; Fig. 10A] and shorter dorsal-fin base (13.1–14.5% of SL vs. 13.9–16.6%; Fig. 10B) and postorbital head length (11.5–12.9% of SL vs. 12.8–14.2%; Fig. 10C).

### ﻿ Stolephorus eldorado sp. nov.

Taxon classificationAnimaliaClupeiformesEngraulidae

8FC6B0AE-0FE2-5383-B73F-DA924E194586

https://zoobank.org/0A916EDA-E70A-4EAF-85C2-7AC51C588BC4

**Figure 11.**
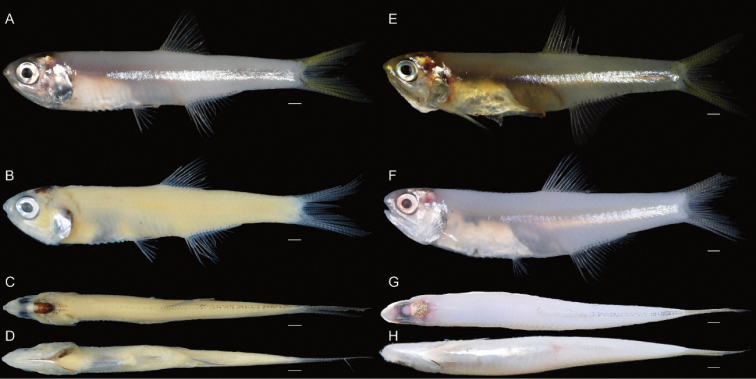
Holotype of *S.eldorado* sp. nov. (KAUM–I. 94517, 44.4 mm SL, Ha Long Bay, northern Vietnam) **A** lateral view (fresh) **B** lateral **C** dorsal, and **D** ventral views (preserved); paratypes of *S.eldorado* sp. nov. (KAUM–I. 67322, 46.7 mm SL, Ha Long Bay, northern Vietnam) **E** lateral view (fresh), (KAUM–I. 110282, 49.5 mm SL, Ke-tzu-liao, southwestern Taiwan) **F** lateral **G** dorsal, and **H** ventral views (fresh). Scale bars indicate 2 mm.

[New English name: El Dorado Anchovy] Figs 1A, B, D, E, F , 11 ; Tables 2 , 3 , 4

Stolephorus insularis (not of Delsman): Whitehead et al. 1988 (in part): 413 unnumbered fig. (Taiwan to Java Sea); Young et al. 1999: 222, fig. 7 (western coast of Taiwan); Wongratana et al. 1999 (in part): 1736, unnumbered fig. (Taiwan to Java Sea); Hata 2018: 41, unnumbered figs (Ha Long Bay, northern Vietnam).
Stolephorus tri (not of Bleeker): Zhang 2001: 129, fig. II-59 (Beihai City, Guangxi Province, China).
Stolephorus bengalensis (not of Dutt and Babu Rao): Hata et al. 2019 (in part): 24, fig. 12a, b (Taiwan; Hainan Island, China; Ha Long Bay, Vietnam; Gulf of Thailand; Songkhla, Thailand; Kuala Terengganu, Terengganu, Malaysia); Hata 2019: 206, unnumbered figs (Ke-tzu-liao, Ziguan District, Kaohsiung, Taiwan); Hata et al. 2022: (in part) 34 (Wenzhou City, Zheijiang Province, China).

#### Holotype.

KAUM–I. 94517, 44.4 mm SL, Ha Long Bay, Ha Long City, Quang Ninh District, Vietnam (purchased at fish market in Ha Long City), 24 Oct 2016; coll. by H. Hata and M. Matsunuma.

#### Paratypes.

57 specimens, 37.5–58.8 mm SL. **Taiwan**: ASIZP 73957, 51.8 mm SL, Fangyan, Changhua (23°57'42.8"N, 120°17'39.8"E); KAUM–I. 110282, 49.5 mm SL, KAUM–I. 113142, 54.0 mm SL, KAUM–I. 113143, 45.5 mm SL, KAUM–I. 113144, 44.3 mm SL, KAUM–I. 113145, 46.3 mm SL, KAUM–I. 113146, 37.5 mm SL, KAUM–I. 113147, 47.3 mm SL, KAUM–I. 113148, 55.3 mm SL, KAUM–I. 113149, 49.4 mm SL, KAUM–I. 113150, 45.9 mm SL, KAUM–I. 113151, 47.3 mm SL, off Ke-tzu-liao, Ziguan District, Kaohsiung. **China**: BMNH 1965.4.1.981–983, 3 specimens, 58.1–58.8 mm SL, Stanley, Hong Kong. **Vietnam**: FRLM 49725, 46.9 mm SL, KAUM–I. 67322, 46.7 mm SL, KAUM–I. 67405, 45.6 mm SL, KAUM–I. 94509, 41.4 mm SL, KAUM–I. 94518, 43.7 mm SL, KAUM–I. 94519, 38.8 mm SL, KAUM–I. 94520, 41.7 mm SL, KAUM–I. 94521, 43.4 mm SL, Ha Long Bay, Ha Long, Quang Ninh Province. **Thailand**: CAS 46931, 8 specimens, 44.4–46.7 mm SL, between Bangsaen and Chol Buri, Chol Buri, Gulf of Thailand; CAS 230414, 4 specimens, 39.9–45.8 mm SL, Lem Nam Point, south tip of Lem Nam Peninsula, Gulf of Thailand (12°02'55"N, 102°35'35"E), approx. 0.6 m depth; KAUM–I. 23190, 48.2 mm SL, Gulf of Thailand (obtained at fish market in Mahachai, Samut Prakan Province), trawl; NSMT-P 142790, 47.9 mm SL, Ko Maeo Island, off Songkhla; URM-P 12398, 3 specimens, 43.0–45.5 mm SL, Song Khula; URM-P 13635, 11 specimens, 46.2–49.5 mm SL, Ang Sila. **Indonesia**: BMNH 1965.10.20.42–47, 6 specimens, 40.6–43.5 mm SL, 20 miles (approx. 32 km) east of Tegal, Java.

#### Diagnosis.

A species of *Stolephorus* with the following combination of characters: 1UGR 16–21 (modally 18), 1LGR 23–28 (25), 1TGR 40–47 (42); 2UGR 10–14 (13), 2LGR 20–24 (23), 2TGR 33–38 (rarely 30) (modally 36); 3UGR 8–12 (modally 10), 3LGR 12–14 (13), 3TGR 20–26 (23); 4UGR 7–10 (8), 4LGR 9–12 (11), 4TGR 16–22 (18); prepelvic scutes 5–7 (6); total vertebrae 38–40 (39); long maxilla, posterior tip just reaching or slightly short of posterior margin of opercle; predorsal scutes present; pelvic scute without spine; body scales deciduous; posterior border of pre-opercle concave, indented; paired dark patch on parietal area with little following pigmentation; distinct double pigment lines along dorsum posterior to dorsal fin; black spots below eye and on lower-jaw tip absent; anal-fin base long, 19.0–22.3% (20.4%) of SL; orbit rather long, 8.2–9.9% (8.9%) of SL; third dorsal-fin ray short, 15.9–18.6% (17.4%) of SL; pelvic fin rather long, 9.1–11.0% (10.0%) of SL, its posterior tip usually not reaching to vertical through dorsal-fin origin when depressed in individuals > 50 mm SL; distance between posterior ends of supramaxilla and maxilla 5.0–6.3% (5.6%) of SL.

#### Description.

Data for holotype presented first, followed by data for paratypes in parentheses (if different). Counts and measurements, expressed as percentages of SL or HL, given in Tables 2 and 3. Body laterally compressed, elongate, deepest at dorsal-fin origin. Dorsal profile of head and body slightly convex from snout tip to dorsal-fin origin, gently lowering to uppermost point of caudal-fin base. Ventral profile of head and body slightly convex from lower jaw tip to pelvic-fin insertion, thereafter, slowly rising to lowermost point of caudal-fin base. Single spine-like scute just anterior to dorsal-fin origin. Abdomen somewhat rounded, covered with six (five to seven) spine-like prepelvic scutes. Pelvic scute without spine. Postpelvic scutes absent. Anus just anterior to anal-fin origin. Snout tip rounded; snout length less than eye diameter. Mouth large, inferior, ventral to body axis, extending backwards beyond posterior margin of eye. Maxilla long, its posterior tip pointed, just reaching (or slightly short of) opercle posterior margin. Lower jaw slender. Single row of conical teeth on both jaws and palatine. Patch of fine conical teeth on pterygoid. Several distinct conical teeth on vomer. Several rows of conical teeth on upper edges of basihyal and basibranchial. Eye large, round, covered with adipose eyelid, positioned laterally on head dorsal to horizontal through pectoral-fin insertion, visible in dorsal view. Pupil round. Orbit elliptical. Nostrils close to each other, anterior to orbit. Posterior margin of pre-opercle concave, indented. Subopercle and opercle with smoothly rounded posterior margins. Gill membrane without serrations. Interorbital space flat, width less than eye diameter. Pseudobranchial filaments present, length of longest filament less than eye diameter. Gill rakers long, slender, rough, visible from side of head when mouth opened. Single row of asperities on anterior surface of gill rakers. Isthmus muscle long, reaching anteriorly to posterior margin of gill membranes. Urohyal hidden by isthmus muscle, not visible without dissection. Gill membrane on each side joined distally, most of isthmus muscle exposed, not covered by gill membrane. Body scales deciduous, completely lacking on all specimens, except for prepelvic scutes. Head scales absent. Fins scaleless, except for broad triangular sheath of scales on caudal fin. Dorsal-fin origin posterior to vertical through base of last pelvic-fin ray, slightly posterior to middle of body. Dorsal and anal fins with three anteriormost rays unbranched. First dorsal- and anal-fin rays minute. Anteriormost three rays of both dorsal and anal fins closely spaced. Anal-fin origin just below base of ninth (eighth to tenth) dorsal-fin ray. Posterior tip of depressed anal fin not reaching caudal-fin base. Uppermost pectoral-fin ray unbranched, inserted below body axis. Posterior tip of pectoral fin not reaching to pelvic fin insertion. Dorsal, ventral and posterior margins of pectoral fin nearly linear. Pelvic fin shorter than pectoral fin, insertion anterior to vertical through dorsal-fin origin. Posterior tip of depressed pelvic fin not reaching to vertical through dorsal-fin origin (reaching to vertical through first to fourth dorsal-fin ray origin in some paratypes smaller than 50 mm SL). Caudal fin forked, posterior tips pointed.

#### Colour of fresh specimens.

(based on colour photographs of KAUM–I. 67322, 46.7 mm SL, KAUM–I. 67405, 45.6 mm SL, KAUM–I. 94517, 44.4 mm SL, KAUM–I. 94521, 43.4 mm SL and KAUM–I. 110282, 49.5 mm SL). Body yellowish milky-white, a silver longitudinal band, of width slightly less than pupil diameter, extending from just above posterior tip of pectoral fin to caudal-fin base. Caudal fin yellow with black posterior margin. Melanophores scattered along caudal-fin rays, ventral surface of caudal peduncle and bases of dorsal and anal fins. Fin rays of dorsal and anal fins yellow. A few melanophores scattered on snout and fin rays of anterior part of dorsal fin. Fin rays and fin membrane of pectoral and pelvic fins transparent whitish, lacking melanophores. A pair of dark patches on parietal region, with little pigmentation on occipital area. Distinct double pigment lines on dorsum from end of dorsal-fin base to caudal-fin base. Body wholly yellowish when freshly caught (Fig. 11E), quickly becoming white after death (Figs 11F–H).

#### Colour of preserved specimens.

Body uniformly pale white. A pair of distinct dark patches on parietal region, with little pigmentation on occipital area. Melanophores scattered on posterior margins of scale pockets on dorsum. Double pigmented lines dorsally posterior to dorsal fin. A few melanophores scattered anteriorly on snout. No black spots below eye and on lower-jaw tip. Melanophores scattered along bases of dorsal and anal fins. All fins transparent, with melanophores scattered along fin rays of caudal fin and anterior parts of dorsal and anal fins.

#### Distribution.

*Stolephoruseldorado* sp. nov. is distributed in the western Pacific from Taiwan to Java, Indonesia (Fig. 4). The species is abundantly caught by trawl and marketed fresh in northern Vietnam. It is a set net bycatch in south-western Taiwan.

#### Etymology.

The specific name “*eldorado*”, referring to the mythical city of gold, reflects the bright yellow colouration of the new species.

#### Morphological comparisons.

*Stolephoruseldorado* sp. nov. has been previously identified as *S.insularis* or *S.bengalensis* (together with *S.bengalensis*, *S.diabolus* and *S.eclipsis* as recognised here) (e.g. Whitehead et al. 1988; Wongratana et al. 1999; Hata et al. 2019). However, *S.eldorado* is distinguished from *S.diabolus* and *S.eclipsis* by having an intermediate number of gill rakers on each gill arch (Table 2; Fig. 7). More detailed comparisons of *S.eldorado* with the latter two species are given in “Comparisons” under each species.

Although *S.eldorado* sp. nov. closely resembles *S.bengalensis* in having very similar numbers of gill rakers on each gill arch, the former differs from the latter in having a greater orbit diameter [maximum orbit diameter 8.2–9.9% (mean 8.9%) of SL vs. 7.3–8.6% (8.1%) in *S.bengalensis* (Fig. 8A)], shorter third dorsal-fin ray [15.9–18.6% (mean 17.4%) of SL vs. 18.5–19.9% (19.0%)] (Fig. 8B) and fewer total vertebrae [38–40 (modally 39) vs. 40 or 41 (40)] (Table 4).

### ﻿Key to species previously identified as *Stolephorusinsularis* by Whitehead et al. (1988) or *Stolephorusbengalensis* by Hata et al. (2019)

**Table:** 

1	1TGR ≤ 38	***S.diabolus* (western coast of Malay Peninsula to Singapore)**
–	1TGR ≥ 41	**2**
2	1TGR ≥ 47; anal-fin base short, less than 19.3% of SL; pelvic fin short, 8.7–9.9% of SL; distance between posterior ends of supramaxilla and maxilla less than 5.4% of SL	***S.eclipsis* (Bintan Island, Indonesia)**
–	1TGR ≤ 47; anal-fin base rather long, more than 19.0% of SL; pelvic fin rather long, 9.1–10.1% of SL; distance between posterior ends of supramaxilla and maxilla more than 5.0% of SL	**3**
3	Third dorsal-fin ray short, 15.9–18.6% (mean 17.5%) of SL; maximum orbit diameter 8.2–9.9% (8.9%) of SL	***S.eldorado* (Taiwan to Java)**
–	Third dorsal-fin ray long, 18.5–19.9% (mean 19.0%) of SL; maximum orbit diameter 7.3–8.6% (8.1%) of SL	***S.bengalensis* (Pakistan to Bay of Bengal)**

## Supplementary Material

XML Treatment for Stolephorus bengalensis

XML Treatment for Stolephorus diabolus

XML Treatment for Stolephorus eclipsis

XML Treatment for Stolephorus eldorado
